# Postpartum Ovarian Vein Thrombosis: Case Report

**DOI:** 10.5811/cpcem.2022.1.53749

**Published:** 2022-04-14

**Authors:** Stephen M. Ferguson, David Arbona, Anthony Furiato

**Affiliations:** HCA Healthcare/University of South Florida Morsani College of Medicine GME/Brandon Regional Hospital, Department of Emergency Medicine, Brandon, Florida

**Keywords:** ovarian vein thrombosis, postpartum, puerperal, hypercoagulability, case report

## Abstract

**Introduction:**

Postpartum ovarian vein thrombosis (POVT) is an uncommon diagnosis that may lead to morbidity or mortality if unrecognized.

**Case Report:**

This report discusses a single case of POVT in a community hospital, along with the treatment and clinical course.

**Conclusion:**

The mechanism is believed to be right-sided clot formation provoked by anatomical and hormonal changes of gestation. Diagnosis is challenging as most patients are previously healthy and symptoms are often vague. Although the differential is broad, modern imaging is sensitive and specific for diagnosis. Prompt treatment with broad-spectrum antibiotics and anticoagulation may reduce morbidity, and prognosis following treatment is excellent.

## INTRODUCTION

Postpartum ovarian vein thrombosis (POVT) is a rare diagnosis most commonly made in the postpartum period. Estimates place its incidence as 0.5–2 cases per 1000 vaginal deliveries, and 20 cases per 1000 cesarean deliveries.^1^,^2^ It was first described in 1956 and has subsequently appeared in case reports and small observational studies.^3^ The pathophysiology of POVT is likely multifactorial, consisting of biochemical and structural alterations.

Pregnancy influences all three elements of Virchow’s triad to augment clot formation. Hormonal changes, including increased estrogen levels, contribute to hypercoagulability. Coagulation factors II, VII, VIII, and X, as well as platelet activating factors and von Willebrand factor, increase during pregnancy. Additionally, protein S and antithrombin III levels decrease and resistance to activated protein C is common.^4^ Endothelial injury occurs due to pro-inflammatory status, direct intrapartum insult, or bacterial endothelial damage.^5^,^6^ Bacterial spread, especially via right ovarian vein from intrauterine sources, may contribute to pathogenesis. Septic thrombophlebitis, considered an infectious variant, depends largely on this spread and the right ovarian vein’s antegrade course.^7^

Gestational venous stasis promotes POVT, while physiological and anatomical variations predispose the right ovarian vein particularly. Approximately 90% of POVT are right-sided. The right gonadal vein follows a longer course, is narrower, and features incompetent valves.^6^,^8^–^10^ Extrinsic factors include dextrorotation compressing the inferior vena cava (IVC) and right ovarian vein, as well as flow reversal during delivery.^2^,^6^,^9^

Known risk factors include prior pro-coagulable state, multiparity, and surgical delivery.^1^,^2^,^10^,^11^ Cesarean section significantly increases POVT risk, especially when combined with underlying coagulopathy.^1^,^2^,^7^,^11^ Some cases concern patients lacking any non-gestational risk factors, however.^9^ Onset is usually within seven days of delivery but may be up to four weeks postpartum.^7^,^9^ The classic presentation is fever, right lower abdominal pain, and a palpable mass. Rarely, however, do patients present with all three elements.^9^ Symptoms are often non-specific, further complicating diagnosis.^2^ Important differential diagnoses include appendicitis, ovarian torsion, endometritis, and tubo-ovarian abscess.

Ovarian vein thrombosis is generally diagnosed via imaging, rather than clinically or surgically.^2^ Ultrasonography, computed tomography (CT), or magnetic resonance imaging (MRI) may be used. Treatment usually consists of antibiotic therapy and months-long or lifetime anticoagulation.^1^,^2^,^7^,^10^,^12^ Antibiotic therapy is usually broad spectrum, with coverage for causative agents in endometritis. Surgical intervention or vena cava filter placement are reserved for rare cases of failed medical management.^1^,^12^

Morbidity in POVT is due to clot extension, sepsis, and intrauterine infection. Pulmonary embolism is the most feared complication and occurs in approximately 13–25% of cases, accounting for much of the 4–5% overall mortality.^6^,^9^,^10^,^12^ Most patients recover without significant morbidity.^13^ This report describes a POVT case identified in the emergency department (ED), exemplifying the diagnostic and therapeutic controversies concerning this rare condition.

## CASE REPORT

The patient was a 20-year-old female who presented to the ED with complaints of right-sided flank and abdominal pain. Her past medical history included idiopathic osteochondromas and benign thyroid nodules. Family history did not elucidate additional risk factors. The patient was primiparous, postpartum day five from vaginal delivery with epidural anesthesia. Term induction of labor was employed due to small gestational size. No other pregnancy complications were reported, apart from second trimester cystitis and third trimester round ligament pain. Clear artificial rupture of membranes was performed; there was no prolonged rupture interval. She tolerated delivery well and was discharged on postpartum day one.

The patient indicated a gradual onset of right lower back pain and lower abdominal pain from postpartum day three onward. She described both pains as “sharp” and positional in nature. She denied prior instances of similar pain, traumatic insult, fever, malodorous vaginal discharge, dysuria, incontinence, or lower extremity paresis. Initial vital signs were as follows: heart rate 119 beats per minute; blood pressure 135/82 millimeters of mercury; oxygen saturation 98% on room air; temperature 37.1° Celsius; and respiration rate of 18 breaths per minute. Examination revealed an uncomfortable, otherwise well-appearing young female with right lumbar tenderness and right suprapubic tenderness.

The differential diagnosis included ascending infection or other causes of sepsis, muscular strain, and post-epidural hematoma or abscess. Contrast CT imaging of lumbar spine, abdomen, and pelvis was obtained. Complete blood count, metabolic panel, lactic acid, and blood cultures were also evaluated. Pain was controlled with intravenous morphine and topical lidocaine. Initial laboratory studies were non-contributory. White blood cell count was 10,400 per microliter (uL) (reference range: 4.5–11.0/uL) without left shift.

**Image 1:**
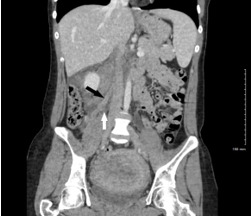
Contrast-enhanced coronal computed tomography image showing tubular enhancing structure along the expected course of right ovarian vein (black arrow) with surrounding fat stranding (white arrow).

**Image 2:**
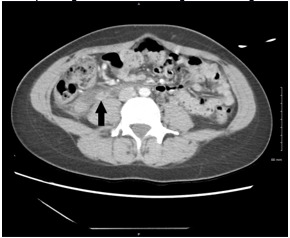
Contrast-enhanced axial computed tomography image showing hyperdense border and lumen corresponding to thrombosis along course of right ovarian vein (black arrow).

The lumbar spine CT showed no mass effect or soft tissue pathology. Abdominal CT demonstrated an “ill-defined tubular structure with extensive fat stranding” following expected right ovarian vein course, terminating in inferior vena cava confluence (IVC) (Images 1 and 2). Subsequent pelvic ultrasound was ordered to further characterize these findings. A complex right adnexal structure was noted but was “indeterminate.”

**CPC-EM Capsule**

What do we already know about this clinical entity?

*Postpartum ovarian vein thrombosis (POVT) represents a life-threatening cause of puerperal pelvic pain. Diagnosis depends on imaging studies and is complicated by non-specific symptoms*.

What makes this presentation of disease reportable?

*Our patient presented with classic symptoms, but lacked established gestational risk factors of pro-coagulable state, multiparity, or surgical delivery*.

What is the major learning point?

*Early recognition, imaging, and treatment of postpartum women presenting with lower abdominal and or pelvic pain can aid diagnosis and reduce morbidity and mortality*.

How might this improve emergency medicine practice?

*The case highlights this rare postpartum differential and diagnostic difficulties, as well as therapeutic uncertainties. This case particularly emphasizes how patients may lack any predisposing factors and the diagnosis merits consideration for postpartum patients presenting to the emergency department*.

Anticoagulation with enoxaparin and empirical antibiotics were initiated. The patient was admitted to the obstetrical service. Hematology consultants recommended IVC ultrasound to better characterize suspected thrombus. Ultrasound confirmed IVC patency, but inadequately visualized culprit ovarian vein. On review of initial imaging, the reading radiologist felt that MRI was not warranted as POVT was highly probable. The patient remained asymptomatic and in stable condition. She was transitioned to oral apixaban and discharged on admission day three.

## DISCUSSION

This case highlights multiple areas of ongoing discussion regarding this rare diagnosis. Given its low incidence, POVT risk factors, clinical presentation, preferred imaging modality, and long-term management are all subjects of debate. Risk factors predisposing POVT, ostensibly absent in this case, may be more common than originally understood. Our patient lacked the conventional peripartum risk factors of cesarean delivery, endometritis, or multiparity. She further lacked personal or family history of hypercoagulability.

Small studies have suggested that many such patients have occult genetic mutations that may not manifest except with the provocation of gestation. For instance, Salomon et al studied 22 patients with POVT, finding that 11 of them had genetic markers for hypercoagulability. The authors went so far as to suggest “prothrombotic tendency” as an element of POVT pathophysiology.^11^ This data demonstrates how outpatient testing for hypercoagulability may aid in prognostication and may guide decisions regarding long-term anticoagulation or future prophylaxis.

In this case, the clinical picture comported with more recent studies on presenting signs and symptoms. The patient had only one element of the classical POVT description comprising fever, pelvic or low abdominal pain, and palpable mass. Furthermore, the patient’s pain was positional and associated with lower back discomfort. Accounting for the patient’s recent epidural anesthesia, the differential broadens beyond even the acute abdomen. Prior to widely available advanced imaging, diagnosis was often made via laparotomy for acute abdomen.^1^ Recent studies describe cases in which the patients lacked specific POVT features or when it was an incidental imaging finding.^2^,^7^,^9^ Jenayah et al found that “only half will experience right lower quadrant abdominal pain.” One study of 50 patients with ovarian vein thrombosis from all causes noted that 18% were discovered incidentally on imaging.^2^ Taken together, the body of literature on POVT implies that diagnosis requires clinical suspicion and low threshold for imaging in equivocal presentations.

Due to diagnostic uncertainty, our patient’s evaluation required multiple imaging modalities, a feature discussed in multiple small studies. For instance, a prospective study of 76 patients with presentations concerning for POVT compared ultrasound, CT, and MRI. In the 12 patients with diagnosed POVT both CT and MRI had sensitivity and specificity of greater than 90%.^14^ In a study of ovarian vein thrombosis in 45 patients from all causes, Wysosinska et al noted that 92% were diagnosed via CT.^15^ Contrast-enhanced CT imaging of the abdomen was employed first in this case. Surrounding fat stranding and tubular mass in the presumed area of ovarian vein course were noted, although images did not demonstrate a clear lumen or clot burden. The original radiologist suggested delayed venous phase imaging, as the contrast load would more clearly delineate the venous system.

Twickler et al used a delayed intravenous contrast load, which may be considered for imaging of suspected POVT. Pelvic ultrasound is commonly employed as a radiation-sparing and cost-effective initial choice, although it was employed for a timely secondary evaluation in this case. Ultrasound lacks sufficient sensitivity (50–56% by some estimates), however, and is often technically limited.^7^,^9^,^10^,^14^ In our case, too, ultrasound proved inadequate for definitive diagnosis due to patient discomfort. Ultrasound of the vena cava was able to ensure patency and exclude clot extension. Contrast-enhanced MRI was considered in this case, but not undertaken. Of note, no reviewed literature involved cases in which MRI discovered POVT that CT failed to detect.

Long-term management of POVT may be the most controversial clinical question regarding the diagnosis. The standard of care includes broad-spectrum antibiotics for presumed endometritis extension and anticoagulation for months, or lifelong.^1^,^10^ One case report cites a 52% mortality without any treatment, with reduction to 5–25% with anticoagulation.^9^ Conversely, multiple articles question anticoagulation’s role, citing limited supporting data.^2^,^13^ Brown et al conducted a randomized intention-to-treat trial of 14 POVT patients. The two study groups were given antibiotics with or without heparin. The results were similar in terms of length of febrile illness and hospitalization. None had continued morbidity or recurrence at three months.^13^

Similarly, Plastini et al reported “no statistically significant correlation found between treatment and no treatment in terms of overall outcomes for patients diagnosed with OVT,” based on a retrospective review of 50 patients with ovarian vein thrombosis from all causes.^2^ The argument against long-term anticoagulation is supported further by radiology-based studies showing thrombus resolution in 7–14 days.^7^ Exceptions to these recommendations are cases of a chronic hypercoagulable state or patients with recurrent thrombosis. These cases likely warrant long-term or gestational anticoagulation.^1^,^4^,^9^

In this case, the patient received one milligram/kilogram enoxaparin twice daily during inpatient anticoagulation. Antibiotics were vancomycin and piperacillin-tazobactam. Antibiotic choice has not been studied, but gentamicin and clindamycin are common choices to cover Gram-positive and anaerobic bacteria. The patient was then transitioned to apixaban. Apixaban was chosen due to its safety and monitoring profile, although it should be noted that this patient did not desire to breastfeed. Novel oral anticoagulants, unlike warfarin, are not recommended in breastfeeding mothers. No trials have compared novel anticoagulants vs warfarin in POVT.^10^ Aspirin is not recommended for prophylaxis.^4^

The recurrence rate of POVT is unknown, but ovarian thrombosis from all causes may recur at rates similar to deep vein thrombosis.^12^,^15^ To date, no recommendations exist regarding prophylaxis in future pregnancy.^1^,^6^ Given this, empirical prophylaxis in future pregnancies is not recommended routinely. Further study via controlled trial or meta-analysis may be an opportunity to study treatment of this rare disease with a sufficiently powered sample.

## CONCLUSION

Postpartum ovarian vein thrombosis is an uncommon, but important, diagnosis for emergency physicians to be familiar with. This unique disorder results from puerperal predisposition to thrombosis, any underlying prothrombotic state, and ascending infection. Diagnosis is difficult, owing to its vague symptoms and a broad differential of more common diagnoses. Imaging with CT is usually sufficient for diagnosis, although other modalities may be required. Treatment consists of anticoagulation to treat thrombosis and antibiotic coverage for a presumed infectious component. Morbidity is due to clot extension or sepsis. The prognosis with treatment is most commonly complete resolution.
